# A population pharmacokinetic model of cabozantinib in healthy volunteers and patients with various cancer types

**DOI:** 10.1007/s00280-018-3581-0

**Published:** 2018-04-23

**Authors:** Steven Lacy, Bei Yang, Jace Nielsen, Dale Miles, Linh Nguyen, Matt Hutmacher

**Affiliations:** 1 Exelixis Inc., 210 East Grand Avenue, South San Francisco, CA 94080-0511 USA; 2 Ann Arbor Pharmacometrics Group, Inc., Ann Arbor, MI USA

**Keywords:** Cabozantinib, Population pharmacokinetics, Cancer types

## Abstract

**Purpose:**

An integrated population pharmacokinetic (popPK) model was developed to describe the pharmacokinetics (PK) of tyrosine kinase inhibitor cabozantinib in healthy volunteers (HVs) and patients with various cancer types and to identify any differences in cabozantinib PK across these populations.

**Methods:**

Plasma concentration data used to develop the popPK model were obtained from nine clinical trials (8072 concentrations from 1534 HVs or patients) of cabozantinib in HVs and patients with renal cell carcinoma (RCC), medullary thyroid carcinoma (MTC), glioblastoma multiforme, castration-resistant prostate cancer, or other advanced malignancies.

**Results:**

PK data across studies were adequately characterized by a two-compartment disposition model with dual first- and zero-order absorption processes and first-order elimination. Baseline demographic covariates (age, weight, gender, race, and cancer type) were generally predicted to have a small-to-moderate impact on apparent clearance (CL/*F*). However, MTC cancer type did show an approximately 93% higher CL/*F* relative to HVs following chronic dosing, resulting in approximately 40–50% lower predicted steady-state cabozantinib plasma concentrations.

**Conclusion:**

This popPK analysis showed cabozantinib CL/*F* values to be higher for patients with MTC and may account for the higher dosage required in this patient population (140-mg) to achieve plasma exposures comparable to those in patients with RCC and other tumor types administered a 60-mg cabozantinib tablet dose. Possible factors that may underlie the higher cabozantinib clearance observed in MTC patients are discussed.

**Electronic supplementary material:**

The online version of this article (10.1007/s00280-018-3581-0) contains supplementary material, which is available to authorized users.

## Introduction

Cabozantinib is a tyrosine kinase inhibitor (TKI) targeting multiple receptor tyrosine kinases implicated in tumor angiogenesis, invasion, and metastasis, including MET (hepatocyte growth factor receptor), VEGFR2 (vascular endolethial growth factor receptor 2), AXL (GAS6 receptor), and RET (glial cell-derived neurotrophic factor receptor) [1]. The cabozantinib capsule formulation (Cometriq^®^) is approved at a dose of 140-mg-free base equivalents (FBE) daily in the USA for the treatment of progressive metastatic medullary thyroid cancer (MTC) and in the European Union (EU) for the treatment of progressive, unresectable locally advanced or metastatic MTC [2, 3]. The cabozantinib tablet formulation (Cabometyx^™^) was subsequently approved at a dose of 60-mg FBE daily in the USA for the treatment of renal cell carcinoma (RCC) following anti-angiogenic therapy and in the EU following prior VEGF-targeted therapy [4, 5]. Cabozantinib tablets are also being evaluated in a pivotal clinical study in patients with hepatocellular carcinoma at a 60-mg FBE daily dose [6].

The cabozantinib tablet formulation (Cabometyx) and capsule formulation (Cometriq) were not bioequivalent following a single 140-mg dose in HVs [7]; the geometric least-squares mean (GLSM) for maximal plasma concentration (*C*_max_) was 19% higher for the tablet formulation and the upper 90% confidence interval for the GLSM ratio for *C*_max_ (131.65%) slightly exceeded the 125% bioequivalence acceptance limit. However, the GLSM values for tablet and capsule formulations were similar (< 10% difference) for both area under the plasma concentration–time curve (AUC_0−*t*_ and AUC_0−∞_) measures, and the 90% CIs were 100–115% around the GLSM ratios.

The 140-mg FBE cabozantinib capsule dose used in the pivotal phase III study in patients with MTC was based on the maximally tolerated dose identified in a phase I study of cabozantinib in patients with MTC and other solid tumors [8]. In the pivotal phase III study, 79% of MTC patients (169 of 214) who received the 140-mg FBE cabozantinib capsule dose eventually dose-reduced [9]. Two protocol-defined cabozantinib dose reductions were allowed: from 140- to 100-mg/day, and from 100- to 60-mg/day. Forty-two percent of MTC patients received 60-mg/day as their final dose [10]. Exposure–response (ER) modeling suggested that the cabozantinib dose reductions from 140- to 100-mg and from 100- to 60-mg were not projected to result in a marked reduction in progression free survival (PFS) or in tumor lesion regrowth in patients with MTC [11, 12].

The 60-mg FBE cabozantinib tablet dose evaluated in the phase III study in patients with RCC was based on findings from a phase I study in patients with RCC of improved tolerance to study drug and evidence of clinical activity in patients who had dose-reduced from 140- to 60-mg [13]. Dose reductions to a 40- or a 20-mg daily dose were permitted in the pivotal phase III study in RCC patients to maintain treatment in response to drug-related adverse events (AEs). Although lower than the 140-mg capsule dose administered to MTC patients, the 60-mg cabozantinib tablet dose was associated with a high percentage of dose reductions in both the phase III study in RCC patients (62%; 206 of 330) [14] and in a phase III study in patients with castrate-resistant prostate cancer (CRPC) (74%; 505 of 682) [15]. ER modeling suggested cabozantinib exposures associated with a simulated 60-mg dose in RCC patients would result in slightly greater decreases in PFS, median percent change of tumor size from baseline, and best overall response rate (based on target lesion) relative to simulated 40- or 20-mg starting doses [16].

A popPK analysis was previously performed on pooled data for 289 cabozantinib-treated cancer patients (including MTC) receiving daily administration of the cabozantinib capsule formulation at a dose of 140-mg FBE/day, except for five subjects that were dosed at 200-mg FBE/day [11]. The data were adequately described by a 1-compartment model with first-order absorption and first-order elimination with a small lag time. The mean CL/*F* and apparent volume of distribution of the central compartment (Vc/*F*) values estimated for a typical White male subject were 4.42 [standard error (SE)%: 2.98%)] L/h and 349 (SE%: 2.73%) L, respectively, resulting in an estimated effective half-life of 55 h. The inter-subject variability (IIV) for CL/*F* (CV%) was 35%.

A popPK analysis of cabozantinib was subsequently performed using data collected from 282 patients with RCC and 63 normal HVs following oral administration of doses of 20-, 40-, and 60-mg [17]. A two-compartment disposition model with dual (fast and slow) lagged first-order absorption processes adequately characterized the concentration–time profile of cabozantinib in HVs and patients with RCC. The mean CL/*F* and terminal-phase volume of distribution (*V*_z_) predicted for a typical White male subject were 2.23 L/h (90% CI 2.13, 2.34) and 319 L (SE%: 2.7%), respectively, resulting in an estimated terminal plasma half-life of approximately 99 h. The IIV for CL/*F* was 46%. These popPK modeling analyses indicated that cabozantinib CL/*F* was approximately twofold lower in RCC patients than in MTC patients, which is consistent with the comparable observed steady-state exposures (*C*_trough,ss_) in RCC and CRPC patients administered a 60-mg tablet dose and in MTC patients administered a 140-mg capsule dose [16]. Based on these apparent differences in cabozantinib PK observed across cancer patients with different tumor types, an integrated popPK model was developed with the pooled PK data from different patient populations and HVs to evaluate the potential impact of patient population, formulations, and doses on the PK of cabozantinib.

## Methods

### Study design and data

The popPK analysis was conducted using data from nine clinical studies of cabozantinib including two phase I studies in HVs [7], a phase I study in cancer patients with advanced malignances [8], phase II studies in patients with GB [18] and CRPC [19, 20], and phase III studies in patients with RCC [14], MTC [9], or CRPC [15]. The results of most of these studies have been previously published; and a summary of the study designs, dosages, and PK sampling schemes is presented in Table 1. All studies were conducted following the ethical principles of the Declaration of Helsinki and Good Clinical Practice guidelines. Written informed consent was obtained from all patients and HVs.

**Table 1:** Summary of clinical studies included in the integrated population pharmacokinetic model of cabozantinib

Study no. (Reference)	Design	Patient population	Cabozantinib dose	Planned pharmacokinetic sampling
XL184-001 [8]	Phase 1, nonrandomized, open-label FIH study	Mixed malignancies	140- or 200-mg	Days 1 and 19: pre-dose, 0.5, 1, 2, 4, 8, and 24 h Day 5: pre-dose and 4 h Days 15 and 29: pre-dose
XL184-010 [7]	Phase 1, crossover BE study of tablet and capsule	Healthy volunteer	140-mg	Pre-dose, 0.5, 1,2,3, 4, 5, 6, 8, 10, 12, 14, 24, 48, 72, 120, 168, 240, 288, 336, 408, and 504 h
XL184-020 [7]	Phase 1, PK of tablet	Healthy volunteer	20-, 40-, 60-mg	Pre-dose, 0.5, 1, 2, 3, 4, 5, 6, 8, 10, 12, 14, 24, 48, 72, 120, 168, 240, 288, 336, 408, and 504 h
XL184-201 [18]	Phase 2, multicenter, open label	Progressive glioblastoma multiforme	140-mg QD	Each Cycle 28 days Cycle 1: pre-dose, and 4 h on Days 1 and 15 Cycle 2: pre-dose and 4 h on Days 29 and 43 Cycle 3 and beyond: pre-dose on day 1
XL184-203 [19, 20]	Phase 2, randomized discontinuation study	Castration-resistant prostate cancer	RDT: 100-mg QD NRE: 40- or 100-mg QD	RDT: pre-dose after “even” weeks after week 12 lead-in, or early termination or adverse event NRE; pre-dose week 1 day 1; pre-dose end of week 3 and 6, 12, 18, and 24, unscheduled, early termination or adverse event
XL184-301 [9]	Phase 3, randomized, double-blind, placebo-controlled	Metastatic medullary thyroid cancer	140-mg QD	Cycle 1, day 1: pre-dose and 2, 4, and 6 h Cycle 2, day 29: pre-dose and 2, 4, and 6 h
XL184-306 [NCT01522443]	Phase 3, randomized, double-blind, controlled versus prednisone	Castration-resistant prostate cancer	60-mg QD	Week 1 day 1, Week 4 day 1 Week 7 day 1, Week 13 day 1
XL184-307 [15]	Phase 3, randomized, double-blind, controlled versus prednisone	Castration-resistant prostate cancer	60-mg QD	End of Week 3 and End of Week 12
XL184-308 [14]	Phase 3, randomized, controlled versus everolimus	Renal cell carcinoma	60-mg QD	Days 29 and 57 approximately eight or more hours after prior evening’s dose

*BE* bioequivalence, *FIH* first-in-human, *QD* once daily, *RDT* randomized discontinuation trial, *NRE* nonrandomized expansion

### Bioanalytical methods

Plasma cabozantinib concentrations were measured using a validated liquid chromatographic-tandem mass spectrometry method. The lower limit of quantitation (LLOQ) was 0.5 ng/mL [21].

### Analysis of data files

Source data in SAS format included information such as PK sample concentrations, PK sample dates and times, dose amounts with dates and times, and patient demographics and covariates. NONMEM^®^ (Version 7) ready data sets were constructed using SAS (Version 9.3), S-plus (Version 8.2) or R (version 3.0.2).

Missing PK drug concentrations, if any, were documented and excluded from the analysis. Drug concentrations which were below the level of quantification (BLQ) were retained in the analysis data set but excluded from the analysis, because the number of BLQ samples was small (< 1%).

Baseline covariate values were assigned using covariate information collected prior to the first dose of study medication. Covariate values closest to the first dose of study medication were used first; however, if covariate information was not available immediately before study drug administration (e.g., pre-dose on day 1), then covariate information from a previous visit (e.g., screening) was used. A summary of the demographic characteristics and relevant covariates for HVs and cancer patients included in the integrated popPK analysis is found in Table 2.

**Table 2:** Baseline demographics and covariates in each clinical study used in the integrated population pharmacokinetic model of cabozantinib

Study	001	010	020	201	203	301	306	307	308	Total
*N* of subjects	40	77	63	39	284	210	41	498	282	1534
Sex										
Male (%)	31 (77.5)	32 (41.6)	33 (52.4)	26 (66.7)	284 (100)	146 (69.5)	41 (100)	498 (100)	222 (78.7)	1313 (85.6)
Female (%)	9 (22.5)	45 (58.4)	30 (47.6)	13 (33.3)	0 (0)	64 (30.5)	0 (0)	0 (0)	60 (21.3)	221 (14.4)
Race										
White (%)	35 (87.5)	74 (96.1)	62 (98.4)	33 (84.6)	246 (86.6)	188 (89.5)	34 (82.9)	380 (76.3)	231 (81.9)	1283 (83.6)
Black (%)	2 (5)	2 (2.6)	1 (1.6)	3 (7.7)	15 (5.3)	1 (0.5)	4 (9.8)	9 (1.8)	5 (1.8)	42 (2.7)
Asian (%)	1 (2.5)	0 (0)	0 (0)	1 (2.6)	14 (4.9)	9 (4.3)	1 (2.4)	1 (0.2)	19 (6.7)	46 (3.0)
Other (%)	2 (5)	1 (1.3)	0 (0)	1 (2.6)	9 (3.2)	5 (2.4)	2 (4.9)	3 (0.6)	16 (5.7)	39 (2.5)
NA (%)	0 (0)	0 (0)	0 (0)	1 (2.6)	0 (0)	7 (3.3)	0 (0)	105 (21.1)	11 (3.9)	124 (8.1)
Population										
Healthy (%)	0 (0)	77 (100)	63 (100)	0 (0)	0 (0)	0 (0)	0 (0)	0 (0)	0 (0)	140 (9.1)
CRPC (%)	0 (0)	0 (0)	0 (0)	0 (0)	284 (100)	0 (0)	41 (100)	498 (100)	0 (0)	823 (53.7)
RCC (%)	0 (0)	0 (0)	0 (0)	0 (0)	0 (0)	0 (0)	0 (0)	0 (0)	282 (100)	282 (18.4)
MTC (%)	0 (0)	0 (0)	0 (0)	0 (0)	0 (0)	210 (100)	0 (0)	0 (0)	0 (0)	210 (13.7)
GB (%)	0 (0)	0 (0)	0 (0)	39 (100)	0 (0)	0 (0)	0 (0)	0 (0)	0 (0)	39 (2.5)
Other^a^ (%)	40 (100)	0 (0)	0 (0)	0 (0)	0 (0)	0 (0)	0 (0)	0 (0)	0 (0)	40 (2.6)
Formulation										
Capsule (%)	40 (100)	75^b^	0 (0)	39 (100)	284 (100)	210 (100)	0 (0)	0 (0)	0 (0)	648 (42.2)
Tablet (%)	0 (0)		63 (100)	0 (0)	0 (0)	0 (0)	41 (100)	498 (100)	282 (100)	959 (62.5)
Body weight										
Range (kg)	53.4–116	46.1–106	58.1–113.5	52–125.3	50.3–182.9	30.4–137.9	57.5–190.7	49.7–140^c^	48.1–155.7	30.4–190.7
Mean (SD)	82.8 (15.9)	71.9 (11.5)	76.4 (11.8)	81.4 (18.3)	90.2 (18.6)	72.9 (18)	89.3 (23.1)	83.3 (14.0)	81.9 (17.0)	82.1 (17.3)
Mean	83.0	71.9	76.5	79.4	87.8	71.3	84.8	82.3	80.4	81.0
Age										
Range (yrs)	23–71	18–55	19–54	20–67	43–87	20–86	48–79	35–87	32–86	18–87
Mean (SD)	56.0 (11.0)	39.3 (9.7)	36.9 (8.6)	48.6 (13.5)	66.3 (8.8)	54.7 (13.3)	64.8 (6.4)	68.7 (7.6)	61.6 (9.5)	61.3 (13.1)
Median	57	39	38	53	67	55	65	69	62	64

*CRPC* castrate resistant prostate cancer, *GB* glioblastoma multiforme, *MTC* medullary thyroid cancer, *N* number, *NA* not available, *RCC* renal cell carcinoma, *SD* standard deviation

^a^Unknown mixed cancer type in Study 001

^b^Study 010 is a cross-over study of capsule versus tablet formulations. The total percentage of subjects on tablet and capsule do not add up to 100% due to due to the crossover design in which each subject received both formulations.

^c^Six subjects in study 307 had missing weight information

### Population PK model

Analyses were performed using the nonlinear mixed effect modeling as implemented in NONMEM (version 7.3 ICON Development Solutions, Ellicott City, MD, USA). Estimation methods used included first-order conditional estimation with interaction (FOCEI), iterative two-stage (ITS), stochastic approximation expectation maximization (SAEM), and importance sampling method (IMP).

#### Base model

The PK Base Model was developed initially using only the exposure data from the HV and RCC studies, and then subsequently with the full integrated data set. Structural models evaluated were one- and two-compartment disposition models with first-order elimination, first-order absorption, and absorption lag time. The previous reports indicated that the concentration–time profile showed multiple peaks suggesting enterohepatic recirculation or multiple absorption sites [7, 21]; therefore, other models were considered such as dual lagged first-order absorption models or transit compartment models with increasing number of transit compartments.

Inter-individual variability (IIV) of the PK parameters was incorporated using a log normal random effects model:

1 $${\theta _{\text{i}}}={\theta _{\text{T}}} \cdot {e^{\left( {{\eta _{\text{i}}}} \right)}},$$

where *θ*_i_ is the individual value of the PK parameter (e.g., CL/*F*), *θ*_T_ is the typical value of the parameter, and *η*_i_ is the inter-individual random effect assumed to have a normal distribution with a mean of zero and variance of *ω*^2^. The vector of IIV random effects had a variance–covariance matrix Ω. A full-block Ω was estimated. Reductions to full-block covariance structure were considered if instability in the model was encountered.

Residual variability (RV), a composite measure of assay error, dose/sample time collection errors, model misspecification, and any other unexplained variability within a subject, was initially modeled using the log-transformed additive error model:

2 $$\ln ({Y_{ij}})=\ln ({C_{ij}})+{\varepsilon _{ij}},$$

where *Y*_*ij*_ denotes the observed drug concentration for the *i*th individual at time *t*_*j*_, *C*_*ij*_ denotes the corresponding predicted concentration based on the PK model, and *ε*_*ij*_ denotes the residual random variable, which is assumed to have a normal distribution with a zero mean and variance *σ*^2^. Other residual error models were explored if patterns were observed in the individual weighted residual (IWRES) versus individual predicted value (IPRED) plot.

#### Covariate model

The covariate analysis was performed using a full model approach [22, 23]. Covariates were pre-specified based upon clinical judgment and mechanistic plausibility and included: age, weight, sex, race, and population (cancer patient type including RCC, CRPC, MTC, GB, and advanced malignancy or HV) on CL/*F* and Vc/*F*. The full model was constructed by including simultaneously all pre-specified covariates of interest into the base model.

The relationship between continuous covariates and the typical value of PK parameters was described using power models:

3 $${\theta _{{\text{TV}},~ij}}~=~{\theta _{{\text{REF}}}}{\left. {\left( {~\frac{{{x_{ij}}}}{{{x_{{\text{REF}}}}}}} \right.} \right)^{{\theta _x}}},$$

where *θ*_REF_ and *θ*_*x*_ are the fixed-effect parameters and *x*_REF_ is a reference value of the covariate *χ*_*ij*_. The approximate median value was used for *χ*_REF_. The relationship between categorical covariate and typical value of PK parameters was modeled as follows:

4 $${\theta _{{\text{TV}},~ij}}={\theta _{{\text{REF}}}} \cdot {\text{exp}}({\theta _x} \cdot {x_{ij~}}),$$

where *θ*_REF_ and *θ*_*x*_ are fixed-effect parameters and *χ*_*ij*_ is the indicator variable with values of 1 or 0. To prevent negative parameter values in simulations, *θ*_*x*_ is the log of the fractional change in the typical value for a categorical covariate.

Plots of the individual random effect values versus covariate values were generated to evaluate if inclusion of the covariate effects reduced or eliminated trends in the random effects/PK parameters. In addition, box plots of the *η* values versus dose and study were generated to evaluate adequacy of pooling studies for this analysis.

At each key step in the model development, a complete battery of diagnostic plots was generated. Standard goodness-of-fit plots were used to assess lack-of-fit. Different structural models were considered if the initial model did not adequately describe the integrated cabozantinib concentration–time data.

#### Posterior predictive check

An internal posterior predictive check (PPC) was performed to assess the predictive performance of the popPK models [24]. A smoothed parametric bootstrap procedure was implemented to account for uncertainty in the parameter estimates. A total of 500 sets of population parameter values were generated using the multivariate normal distribution with the mean vector set to the population parameter estimates and the covariance matrix set to that of the final model. These values were used to simulate a data set replicating study design, sample size, and covariate distributions from the observed data set. The PPC statistics including the median, and 10th and 90th percentiles were computed at nominal time points for both the observed and each simulated data set. Prediction intervals were constructed based on the 5th and 95th percentiles of the simulated distributions of the PPC statistics.

## Results

### Data

The final data set contained a total of 8072 plasma concentrations from 1534 patients and HVs. All BLQ samples were excluded from the analysis, since the percentage of samples that were BLQ was small (< 1%).

The majority of subjects were male (85.6%), as four of the nine studies enrolled patients with CRPC, and white (83.6%). Body weights and ages were generally consistent across the studies, except for the HV studies which had younger subjects due to the exclusion criteria. The single-dose clinical pharmacology studies in HVs and the phase I safety study that enrolled patients with advanced mixed malignancies (XL184-001) contained intensive sampling which allowed for full characterization of the cabozantinib PK profile. The phase II and III clinical studies in cancer patients administered cabozantinib daily provided sparse PK sampling data.

### Population pharmacokinetic modeling

Supplemental Table 1 lists the key steps in development of the popPK model. Initially, the base popPK model was developed utilizing only exposure data from studies enrolling HVs or RCC patients. A two-compartment disposition model and a dose-dependent dual absorption model containing two lagged first-order absorption processes (dose-dependent fast and slow) adequately characterized the cabozantinib PK data. However, numerical problems and long run times were encountered when the model was fit to the fully integrated data set that contained all study populations and covariates [including demographics (age, weight, sex, and race) and cancer population effects (RCC, CRPC, MTC, GB, and advanced malignancies)] (full model). Therefore, the dual first-order absorption model was replaced by a model with dual first-order and zero-order absorption [full modified (FM) model]. The first-order absorption process including a lag time and a dose-dependent effect on the absorption rate constant (Ka) was described using a power model. In addition, capsule formulation was included as a structural covariate on Ka and overall bioavailability based on prior knowledge from the capsule-tablet bioequivalence study (XL184-020; [7]).

**Fig. 1:**
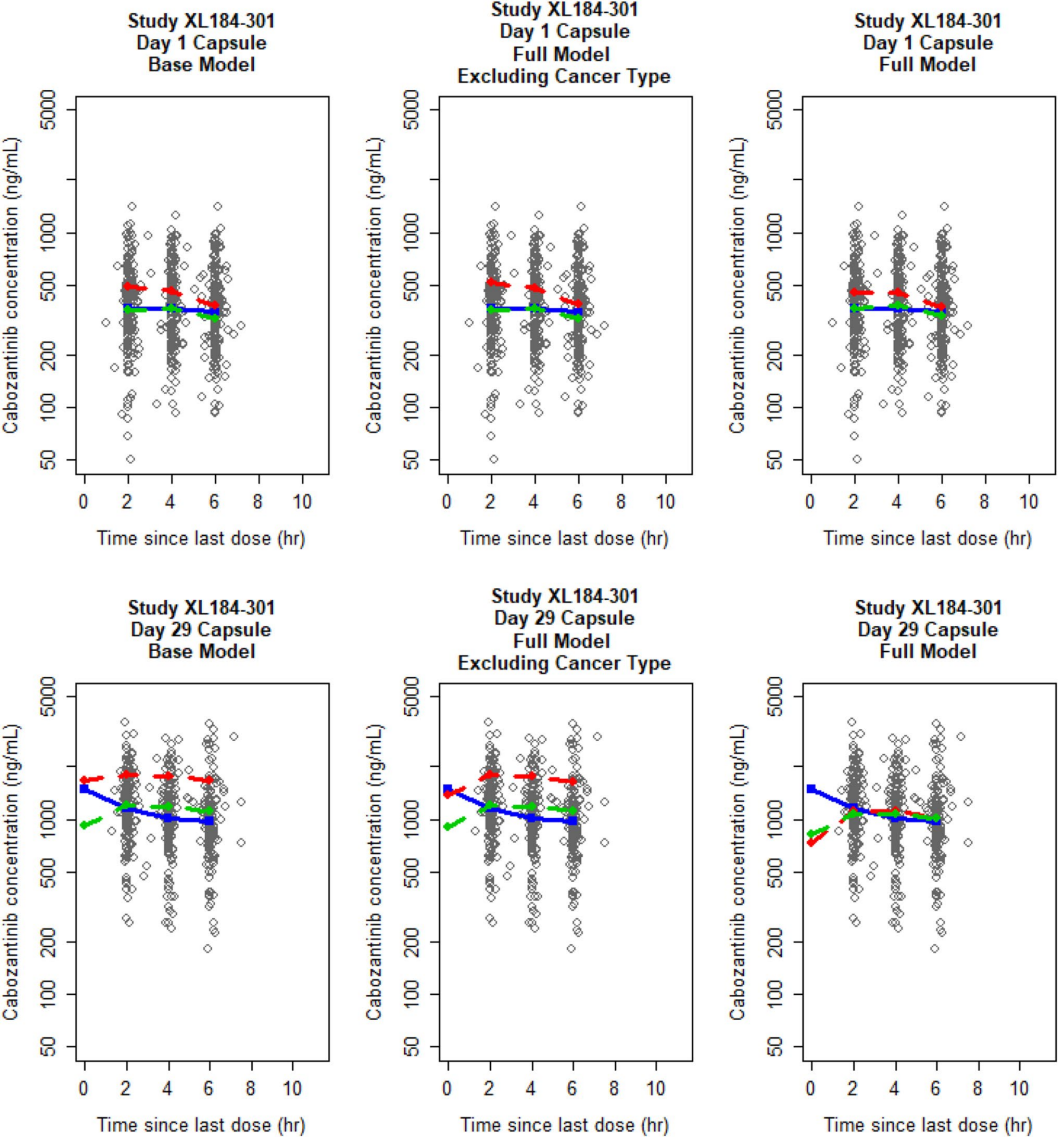
Comparison of goodness-of-fit plots for patients with medullary thyroid cancer on day 1 and day 29 of study XL184-301. Solid blue, red-dashed, and green-dashed lines correspond to geometric mean observed, typical individual predicted (PREDs), and individually predicted (IPREDs) concentrations, respectively

**Fig. 2:**
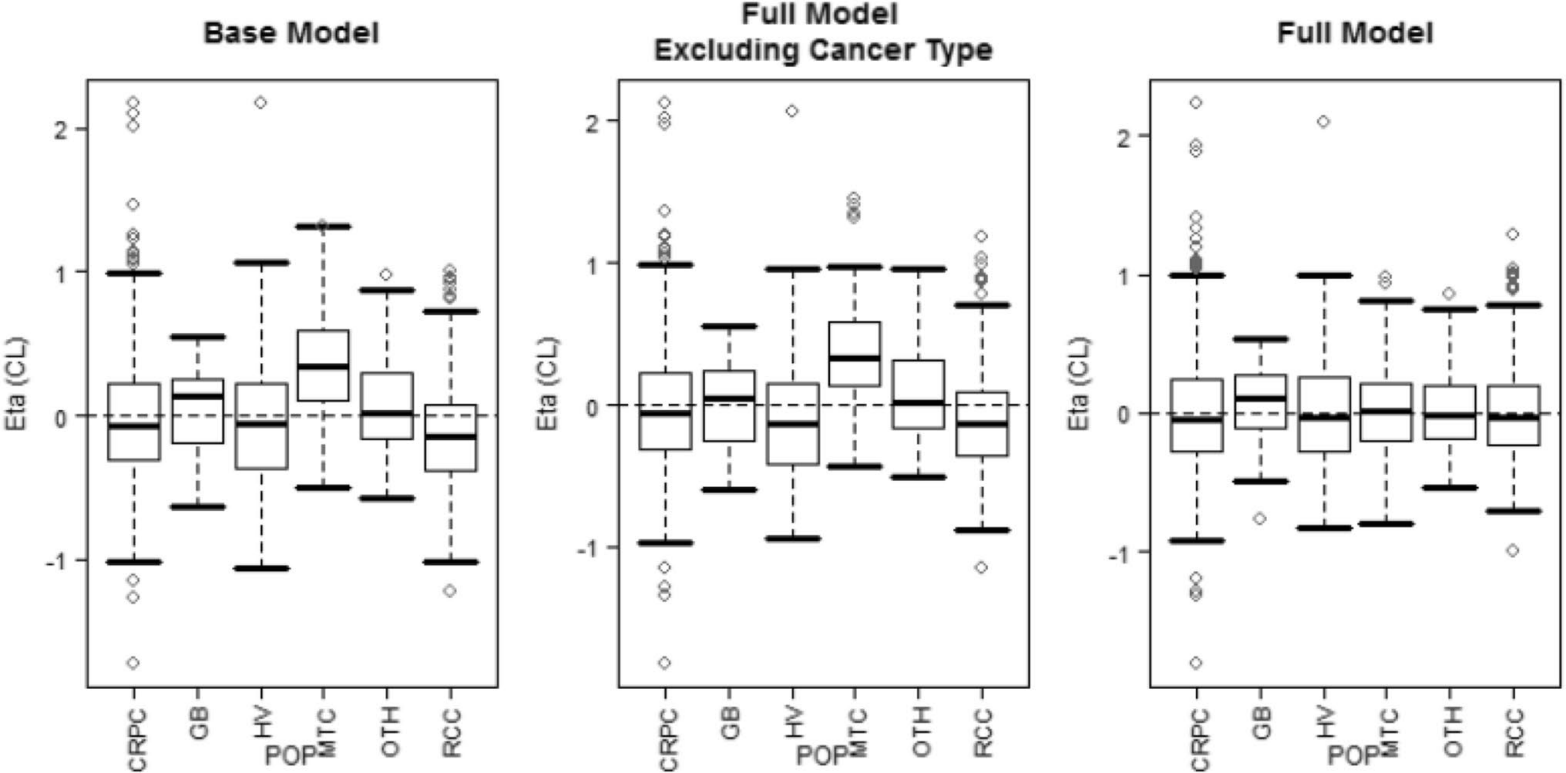
Inter-individual random effect (Eta) on CL/*F* versus subject population. The boxes represent median and 25th and 75th percentiles. The bars represent 5th and 95th percentiles The open circles represent individual values outside the 5th and 9th percentiles. *CL* clearance, *CRPC* castrate-resistant prostate cancer, *GB* glioblastoma multiforme, *HV* healthy volunteer, *MTC* medullary thyroid cancer, *OTH* other cancer types in Study XL184-001, *POP* population, *RCC* renal cell carcinoma

The FM model was stable and adequately described the PK data in the fully integrated cabozantinib PK data set across different studies (Supplemental Fig. 1). Close inspection of the model output suggested that the magnitude of demographic and cancer population-specific covariate effects on cabozantinib PK was small, except for MTC patient population who had a substantially higher estimated CL/*F*. To determine the significance of covariate effects, and the MTC covariate effect on CL/*F*, two additional ad hoc model runs were performed relative to the FM model: (1) all covariates except for dose on k12 and capsule on Ka and F1 were removed (BASE) and (2) cancer-type covariates on CL/*F* and Vc/*F* were removed (FMECT). The OFV was increased 401.2 units when all covariates (i.e., demographic and cancer type) were removed (comparing BASE to FM), suggesting significant effects of demographic covariates and cancer types together. The OFV was 305.9 units higher when cancer-type covariates were excluded (comparing FMECT to FM), indicating a significant effect of one or more cancer types on the PK of cabozantinib. Furthermore, the OFV for BASE model was 95.4 units higher than the OFV for the FMECT model, suggesting that some other demographic covariates were also significant, but their effect was less than cancer-type covariates. Goodness-of-fit plots showed all three models provided reasonable fit to the data, but there was some lack-of-fit between observed and predicted geometric means concentrations for MTC patients for the BASE and FMECT models, with MTC patients having a higher estimated CL/*F* (Fig. 1). Only after including cancer type as a covariate on CL/*F* (FM model) did the trend between IIR on CL/*F* and cancer patient population resolve (Fig. 2). Thus, the FM model was determined to be the final model.

**Fig. 3:**
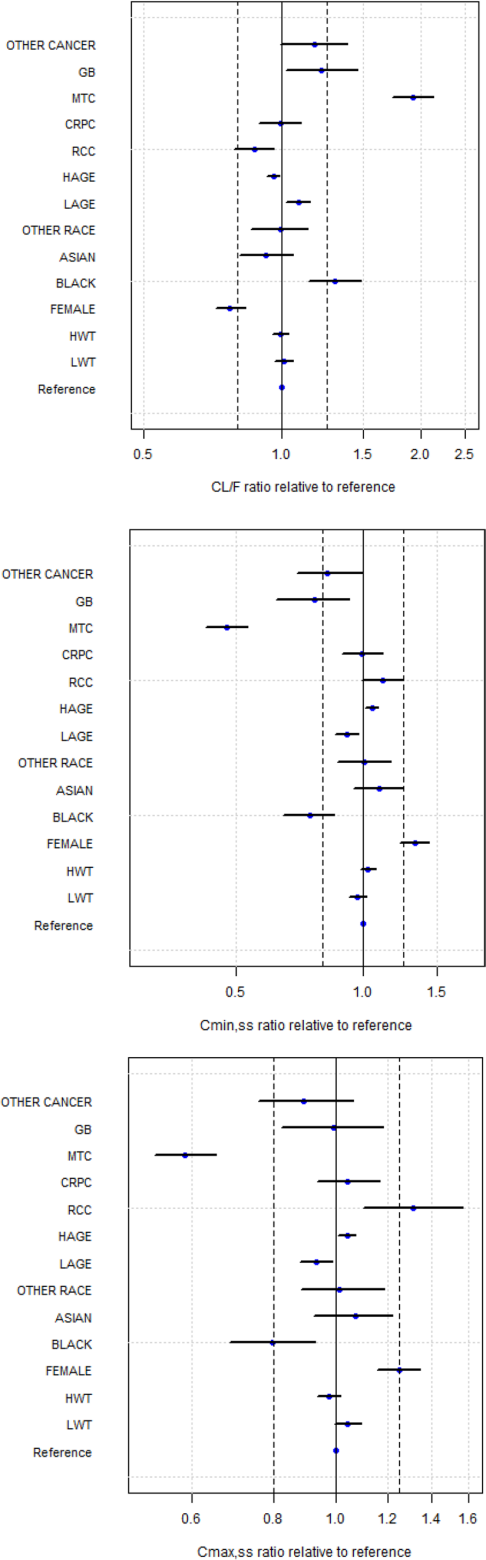
Impact of covariates on steady-state cabozantinib CL/*F, C*_min_ and *C*_max_ relative to a reference White, male, 80 kg, 60 year-old healthy subject. CL/*F*, apparent clearance, *Cmax,ss* maximum plasma concentration at steady state, *Cmin,ss* minimum plasma concentration at steady state, *CRPC* castrate-resistant prostate cancer, *GB* glioblastoma multiforme, *HAGE* a 79-year-old subject, *HWT* a subject with body weight of 112 kg, *LAGE* a 36-year-old subject, *LWT* a subject with body weight of 56 kg, *MTC* medullary thyroid cancer, *RCC* renal cell carcinoma

Cabozantinib PK parameter estimates for all three models are shown in Table 3. For the FM model, the transformed estimates (90% CI) for CL/*F* and Vc/*F* were 2.478 (2.257, 2.721) L/h and 187 (156.3, 223.9) L, respectively. Demographic covariates (age, weight, sex, and race) generally showed minimal effect on CL/*F* and Vc/*F*, although race covariate Black did result in an approximately 30% increase in CL/*F*. Cancer-type covariates RCC, CRPC, GB and Other showed minimal effects on CL/*F* and Vc/*F*, whereas patients with MTC cancer type were predicted to have approximately 93% higher CL/*F* relative to HVs. Thus, when compared to HVs at the same dosage, patients with MTC would have approximately 40 and 50% lower steady-state maximal (*C*_max,ss_) and minimal (*C*_min,ss_) exposures, respectively (Fig. 3).

**Table 3:** Parameter estimates for the final integrated population pharmacokinetic model of cabozantinib in patients with different cancer types

Parameter	Base model (BASE)	Full model excluding cancer type (FMECT)	Full model (FM)
	Transformed Estimate (90% CI)	Transformed estimate (90% CI)	Transformed estimate (90% CI)
Ka (h^− 1^)	0.804 (0.576, 1.123)	0.846 (0.606, 1.182)	0.979 (0.679, 1.411)
Duration of absorption for the zero-order absorption process (h)	2.435 (1.966, 3.016)	2.441 (92.096, 2.843)	2.4 (2.01, 2.866)
Cl/*F* (L/h)	2.457 (2.396, 2.519)	2.553 (2.482, 2.625)	2.478 (2.257, 2.721)
Vc/*F* (L)	157.178 (142.879, 172.95)	146.713 (131.894, 163.204)	187.0 (156.3. 223.9)
Q/*F* (L/h)	30.154 (27.743, 32.786)	30.118 (27.883, 32.525)	31.213 (28.732, 33.92)
Vp/*F* (L)	188.666 (176.091, 202.148)	193.605 (182.546, 205.203)	195.1 (183.3, 207.9)
ALAG1 (h)	0.789 (0.763, 0.815)	0.777 (0.752, 0.804)	0.784 (0.757, 0.812)
Fraction of dose in the first absorption depot F1^a^	0.847 (0.805, 0.881)	0.840 (0.803, 0.8720)	0.854 (0.819, 0.884)
Dose-dependent Ka^c^	0.566 (0.199–0.934)	0.585 (0.201, 0.969)	0.677 (0.268, 1.085)
Covariates			
Capsule on Ka^b^	− 0.211 (− 0.541, 0.354)	− 0.300 (− 0.599, 0.224)	− 0.579 (− 0.783, − 0.183)
Capsule on overall relative oral availability^b^	− 0.189 (− 0.205, − 0.173)	− 0.183 (− 0.199, − 0.167)	− 0.144 (− 0.162, − 0.126)
Age on CL/*F*^c^		− 0.273 (− 0.367, − 0.178)	− 0.162 (− 0.281, − 0.042)
Female on CL/*F*^b^	–	− 0.233 (− 0.29, − 0.172)	− 0.230 (− 0.286, − 0.17)
Race (Black) on CL/*F*^b^	–	0.249 (0.085, 0.439)	0.301 (0.139, 0.486)
Race (Asian) on CL/*F*^b^	–	− 0.118 (− 0.233, 0.013)	− 0.078 (− 0.192, 0.052)
Race (Other) on CL/*F*^b^	–	− 0.029 (− 0.161, 0.124)	− 0.007 (− 0.0136, 0.414)
Weight on CL/*F*^c^	–	− 0.248 (− 0.373, − 0.122)	− 0.028 (− 0.148, 0.092)
RCC on CL/*F*^b^	–	–	− 0.129 (− 0.217, − 0.033)
CRPC on CL/*F*^b^	–	–	− 0.009 (− 0.11, 0.103)
MTC on CL/*F*^b^	–	–	0.928 (0.738, 1.136)
GB on CL/*F*^b^	–	–	0.216 (0.02, 0.449)
Other malignancies on CL/*F*^b^	–	–	0.178 (0.003, 0.384)
Age on Vc/*F*^c^		− 0.277 (− 0.459, − 0.095)	− 0.012 (− 0.247, 0.223)
Female on Vc/*F*^b^	–	0.165(0.023, 0.327)	0.11 (− 0.033, 0.276)
Race (Black) on Vc/*F*^b^	–	− 0.065 (− 0.362, 0.372)	− 0.022 (− 0.334, 0.438)
Race (Asian) on Vc/*F*^b^	–	0.125 (− 0.197, 0.576)	0.05 (− 0.278, 0.528)
Race (Other) on Vc/*F*^b^	–	− 0.018 (− 0.321, 0.422)	− 0.059 (− 0.382, 0.435)
Weight on Vc/*F*^c^	–	0.798 (0.513, 1.083)	1.019 (0.72, 1.318)
RCC on Vc/*F*^b^	–	–	− 0.63 (− 0.853, − 0.069)
CRPC on Vc/*F*^b^	–	–	− 0.241 (− 0.395, − 0.049)
MTC on Vc/*F*^b^	–	–	− 0.07 (− 0.232, 0.125)
GB on Vc/*F*^b^	–	–	− 0.569 (− 0.72, − 0.337)
Other malignancies on Vc/*F*^b^	–	–	− 0.186 (− 0.372, 0.055)
Variance^d^	–	–	–

*ALAG*, absorption lag time, *CI* confidence interval, *CL*/*F* apparent clearance, *CRPC* castrate-resistant prostate cancer, *F1* fraction of dose split to the first absorption depot in a dual absorption model, *GB* Glioblastoma multiforme, *Ka* absorption rate constant, *MTC* medullary thyroid cancer, *Q*/*F* apparent flow parameter between compartments, *RCC* renal cell carcinoma *Vc*/*F* apparent volume of distribution of the central compartment, *Vp*/*F* apparent volume of distribution of the peripheral compartment

^a^Anti-logit transformation was used to obtain F1

^b^For categorical covariates (e.g., capsule), transformed estimates correspond to fractional change from the reference level

^c^untransformed values

^d^Untransformed full model variance estimates (90% CI) *σ*2 = 0.118 (0.114, 0.122); *ω*^2^_Ka = 2.063 (1.579, 2.548); *ω*^2^_CL/*F* = 0.202 (0.185, 0.218); *ω*^2^_Vc/*F* = 0.233 (0.193, 0.273); *ω*^2^_F1 = 0.466 (0.385, 0.546); *ω*^2^_CL/*F*:Vc/*F* = 2.475 (1.923, 3.028), where *ω*^2^ is the variance–covariance matrix (Ω) of the inter-individual random effects (*η*) in the pharmacokinetic parameter, and *σ* the variance–covariance matrix of the intra-individual random effects (*ε*) in the measurements

The predictive performance of the three models for patients with MTC stratified by day of study demonstrated that the lack-of-fit was most apparent on day 29 of the study for the BASE and FMECT models (Supplemental Fig. 2). These findings suggested that the day 29 concentration data and reduced accumulation relative to that expected from HVs are largely responsible for driving the increase in CL/*F* for patients with MTC. To confirm this finding, an additional ad hoc run was performed using the FM model to a re-fit data set including only day 1 data. In this model, the population effect for MTC was − 0.312 (90% CI − 0.824, 0.201), suggesting that CL/*F* on day 1 for MTC patients was not significantly different from HVs. Model prediction using this ad hoc model run remained reasonable for PK data on day 1, but, when the predictions from the ad hoc model were applied to day 29, the simulated data were much higher than observed PK data for MTC patients (Supplemental Fig. 3). Using the full data set and the FM model which included a covariate for MTC patients, the fit was substantially improved on day 29, while an acceptable fit was maintained on day 1 (Supplemental Fig. 2).

## Discussion

Cabozantinib is a TKI approved for the treatment of MTC and RCC [2–5]. Although the formulations and dosages are different for MTC (140-mg/day Cometriq) and RCC (60-mg/day Cabometyx) and dose adjustments and interruptions were allowed in the respective phase III studies, the resultant cabozantinib steady-state exposures in the pivotal phase III studies were comparable for the two patient populations [16]. Findings from popPK analyses subsequently showed that cabozantinib CL/*F* (CV%) in MTC patients [4.4 L/h (35%)] was twofold higher than in RCC patients [2.2 L/h (46%)] [11, 17], suggesting an apparent difference in cabozantinib clearance in patients with different tumor types.

To examine the extent to which demographic covariates could explain heterogeneity in the PK parameters across cancer populations, an integrated population PK analysis of cabozantinib was conducted using exposure data from HVs and cancer patients with different types of malignancies (ie, MTC, RCC, CRPC, GB). This analysis included data from nine clinical studies (three phase I, two phase II and four phase III) for a total of 8072 cabozantinib concentration records from 1534 subjects. A two-compartment model with first-order elimination and a dual absorption (first-order + zero-order) process adequately described the observed cabozantinib PK data.

The FM model which incorporated demographic covariates (age, body weight, sex, and race) and type of cancer malignancy (RCC, CRPC, MTC, GB, and other malignancies) on cabozantinib CL/*F* and Vc/*F* was evaluated. While most covariate effects (including patient demographics) included in the FM model had small-to-moderate effects on cabozantinib PK parameters and exposure metrics, MTC cancer-type led to a > 90% increase in CL/*F*. Ad hoc analyses showed that the cabozantinib concentrations at day 29 were primarily driving the increase in CL/*F* in MTC patients in pivotal phase III study XL184-301. MTC patients had lower steady-state plasma concentrations at day 29 than anticipated for the given dose relative to patients with other cancer types or HVs and suggested that lower observed accumulation could be due to higher clearance in this patient population. Possible reasons for the large increase in cabozantinib clearance at steady state for MTC patients evident in the integrated popPK analysis were explored, including differences in treatment-emergent AEs, concomitant medications, and administered cabozantinib dose.

Diarrhea is a common treatment-related AE in cancer patients receiving cabozantinib [8, 9, 13–15], and the 140-mg dose administered to MTC patients may be anticipated to result in a higher incidence and/or severity of diarrhea than a 60-mg dose given to RCC and CRPC subjects. As cabozantinib is considered to undergo enterohepatic recirculation [21], a decrease in the absorption fraction of cabozantinib typically reabsorbed via enterohepatic reabsorption due to treatment-related diarrhea may result in an apparent increase in cabozantinib clearance. The severity of diarrhea and possible effect on clearance would be anticipated to be greater in MTC patients administered a higher cabozantinib dose (140-mg) than that given to patients with other tumor types (60-mg). However, there was no marked difference in Grade 3/4 diarrhea in the subjects enrolled in the cabozantinib arm of the pivotal MTC study administered a 140-mg dose [16% (34 of 214); [9]] and in the cabozantinib arm of the pivotal RCC study administered a 60-mg dose [13% (43 of 311); [14]].

In a separate popPK analysis [26], MTC patients were reported to have higher (67% greater) oral clearance for another TKI (motesanib) relative to patients with differentiated thyroid cancer (DTC), in conjunction with a higher baseline incidence rate of diarrhea (68 and 6% in MTC and DTC cohorts, respectively). Similar to cabozantinib, patients’ disease type best accounted for inter-patient variability in motesanib CL/*F* of all covariates tested. However, incorporating diarrhea into the popPK model did not result in a significant improvement in the model fit, after accounting for the patients’ disease type, and there was no difference in motesanib CL/*F* among MTC patients with severe, moderate, and mild diarrhea. In addition, both the motesantib and cabozantinib popPK analyses showed a minimal effect on Vc/*F* in MTC patients, whereas a reduction in oral bioavailability due to diarrhea would be expected to result in increases in both CL/*F* and Vc/*F*. The mechanistic basis for the difference in motesanib CL/*F* between DTC and MTC patients was not identified.

Increased cabozantinib clearance in MTC patients at steady state could be related to treatment-emergent hypocalcemia, particularly in advanced MTC patients who undergo thyroidectomy when the parathyroid glands are also partially or completely removed resulting in decreased plasma parathyroid hormone levels. Hypocalcemia may affect drug clearance indirectly via stimulation of active vitamin D metabolite 1,25 dihydroxyvitamin D_3_ (1,25(OH)_2_D_3_) synthesis, and subsequent induction of CYP3A4 by 1α,25(OH)_2_D_3_ [27, 28]. Since cabozantinib is metabolized by CYP3A4 [29], hypocalcemia was considered as a potential contributing factor in reducing cabozantinib clearance in MTC patients. Although clinical laboratory-defined hypocalcemia was identified in 52% of MTC patients receiving cabozantinib in the pivotal phase III study XL184-301 [2], and in fewer MTC patients receiving placebo in the same study (27%), overall no evidence of altered calcium levels was noted in patients with MTC compared to other cancers to suggest that hypocalcemia was responsible for increased cabozantinib clearance in this population.

Differences in concomitant medication use, particularly administration of strong CYP3A4 inducers in MTC patients, could have resulted in the increased cabozantinib clearance observed in the MTC patient population. However, only 1.4% of patients (3 of 207 total) were reported to have used a concomitant strong CYP3A4 inducer in the MTC phase III study of cabozantinib [11]. Cabozantinib is also a substrate of efflux transporter MRP2 [25], so concomitant administration of an MRP2 inducer could potentially increase cabozantinib clearance by enhancing hepatic and/or intestinal drug MRP2-mediated transport activity. Although overall use of concomitant MRP2 inducers was not documented for MTC patients in study XL184-301, only 5.5% of MTC subjects (12 of 219) administered cabozantinib were reported to have received MRP2 inducer (and moderate CYP3A4 inducer) dexamethasone.

Cabozantinib plasma clearance (CL/*F*) may also appear to be higher if oral bioavailability (*F*) decreased with increasing cabozantinib dose. The approved cabozantinib dose for MTC patients (140-mg) is higher than the dose approved for RCC patients and dose generally administered to non-MTC patient populations (60-mg), and steady-state CL/*F* in the MTC popPK analysis (4.4 L/h) was higher than that determined in the RCC popPK analysis (2.2 L/h). However, no decrease in cabozantinib oral bioavailability was evident in a cross-study analysis indicating generally dose-linear PK for tablet and capsule formulations over a broad dose range (20–140 mg) [25]. In addition, lower cabozantinib exposures associated with higher relative CL/*F* in MTC patients were only observed at steady state (day 29) and not at day 1.

Alternatively, estimates of CL/*F* values from MTC subjects that tolerate a 140-mg daily cabozantinib dose may be higher than the overall study population if they reflect a sub-population that tolerates this higher dose at steady state based on a faster relative intrinsic clearance. In the MTC popPK analysis, high drop-out rate or early discontinuation was also considered to possibly explain the lower day 29 concentrations in MTC patients relative to HVs [11]. If subjects with low clearance and higher exposures dropped out or discontinued the study early due to treatment-emergent AEs, only those subjects with higher clearances resulting in lower, more tolerable exposures would remain. This scenario is unlikely considering 79% of the patients in the MTC popPK analysis contributed PK samples on both days 1 and 29.

Finally, a more detailed PK sampling of the terminal elimination phase was included in the RCC popPK analysis (up to 504-h post-dose in HVs) than in the MTC popPK analysis where the final PK sample was taken at approximately 24-h post-dose. As cabozantinib has a relatively long plasma terminal half-life (HV mean: 118 h [25]), plasma clearance could have been underestimated in the MTC popPK analysis based on a more limited PK data collection of terminal elimination phase. However, the integrated popPK model developed subsequently included exposure data from patients with different tumor types and HVs; the addition of all covariates, including demographic (age, weight sex, and race) and population (RCC, CRPC, MTC, GB, and advanced malignancies) on both CL/*F* and Vc/*F* resulted in an adequate fit to the data. The magnitude of most demographic and population-specific covariate effects on cabozantinib PK was small, except for MTC patient population who had a substantially larger estimated cabozantinib CL/*F*. Thus, model-related and PK sampling differences do not appear to underlie the higher CL/*F* values in MTC patients evident at steady state.

## Conclusion

In summary, results from the integrated popPK analysis indicate that compared with other cancer patient groups (RCC, CRPC, and GB), MTC patients clear cabozantinib faster and thus have lower dose-normalized steady-state plasma exposures. Cabozantinib PK appears to be time-varying in MTC patients, as day 1 CL/*F* values were lower and comparable to those in non-MTC cancer patient populations. Several possible factors may underlie the higher cabozantinib clearance observed in MTC patients; however, an exact cause has yet to be identified. Based on the integrated popPK analysis, non-MTC cancer patient cohorts (including RCC patients) appear to have comparable cabozantinib clearance to that of HVs.

## Electronic supplementary material

Below is the link to the electronic supplementary material.

Supplemental Fig. 1 Goodness-of-fit plots for the FM Model. Blue, red, and green lines correspond geometric mean observed, typical individual predicted (PREDs), and individually predicted (IPREDs) concentrations, respectively. (DOCX 122 KB)

Supplemental Fig. 2 Posterior Predictive Check: 90% Prediction Intervals and Observed Geometric Means, 10^th^ and Percntiles of Cabozantinib Concentrations versus Time Profiles by Study for Patients with MTC. Squares, circles, and triangles correspond to observed median, 10^th^, and 90^th^ percentiles, respectively. Middle, lower, and upper shaded areas correspond to 90% prediction intervals for median, and 10^th^ and 90^th^ percentiles, respectively. *MTC* medullary thyroid cancer (DOCX 111 KB)

Supplemental Fig. 3 Visual Predictive Check for Cabozantinib Concentrations in Patients with MTC Using Full Model Re-Fit Including Only day 1 Data. Squares, circles, and triangles correspond to observed median, and 10^th^, and 90^th^ percentiles, respectively. Middle, lower, and upper shaded areas correspond to 90% prediction intervals for median, and 10^th^ and 90^th^ percentiles, respectively. *MTC* medullary thyroid cancer (DOCX 54 KB)

Supplementary material 4 (DOCX 17 KB)
