# Supplementary material for: A population pharmacokinetic model of cabozantinib in healthy volunteers and patients with various cancer types
Source: Cancer Chemother Pharmacol. 2018 Apr 23;81(6):1071–82. doi: 10.1007/s00280-018-3581-0 (PMC5973963; doi:10.1007/s00280-018-3581-0)
Supplement: Supplementary file 1 — Supplemental Fig. 1 Goodness-of-fit plots for the FM Model. Blue, red, and green lines correspond geometric mean observed, typical individual predicted (PREDs), and individually predicted (IPREDs) concentrations, respectively. (DOCX 122 KB) [file 280_2018_3581_MOESM1_ESM.docx]

**Supplemental Fig. 1**


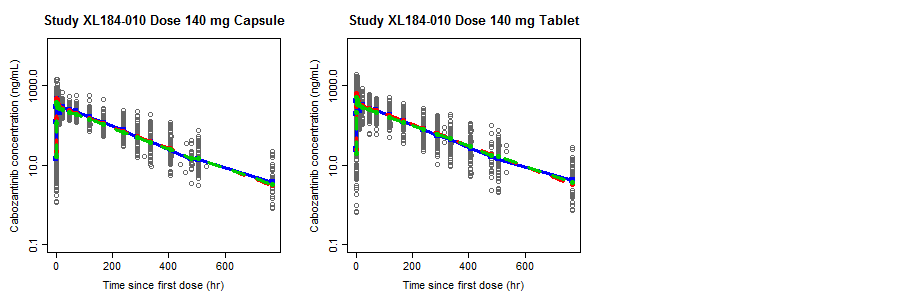


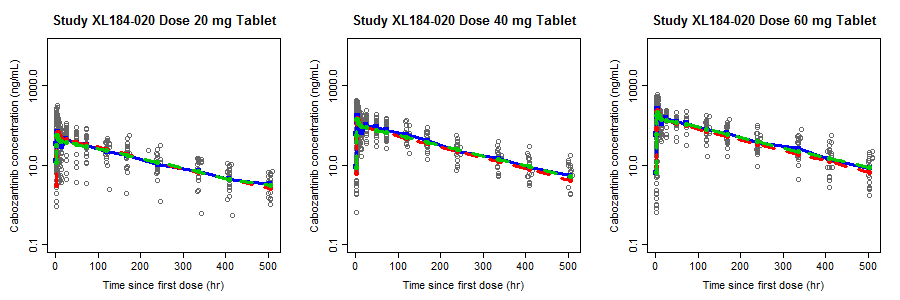


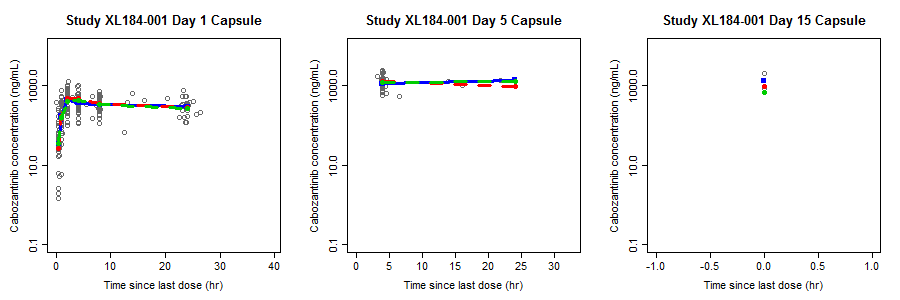


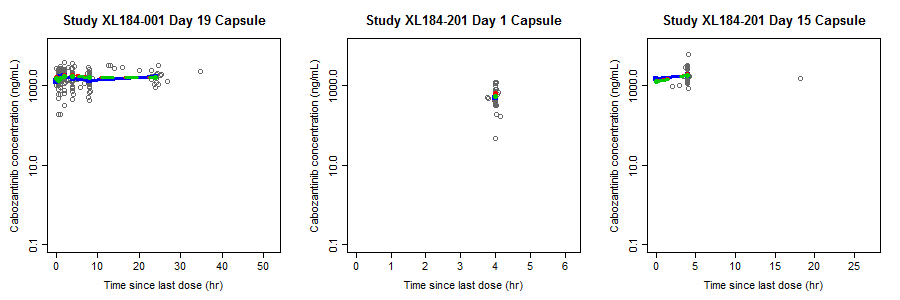


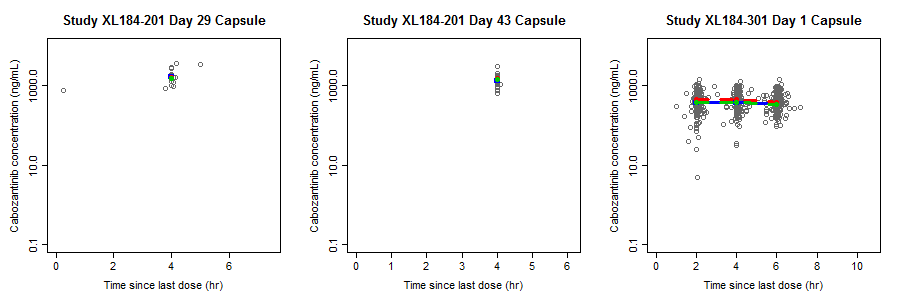


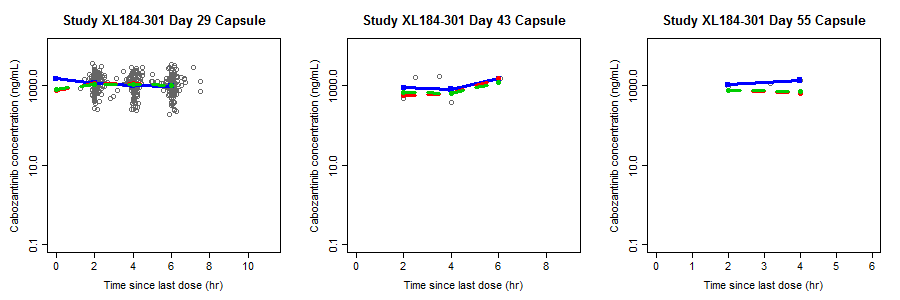


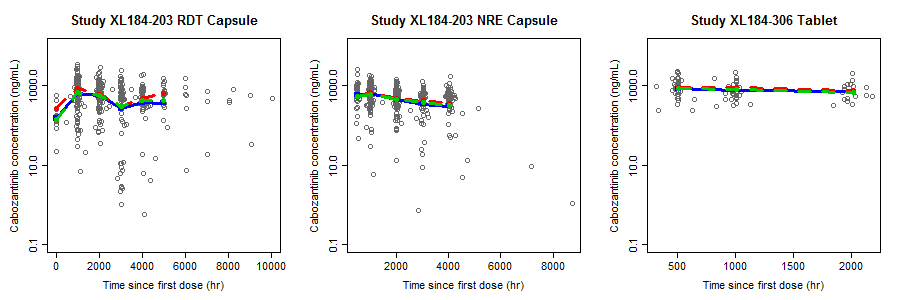


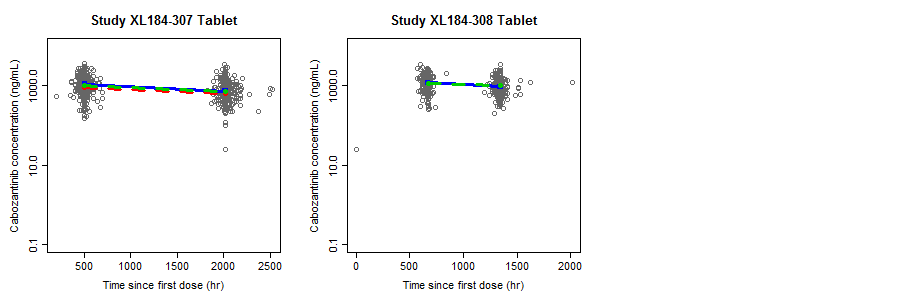


**Goodness-of-Fit Plots for the FM Model.** Blue, red and green lines correspond geometric mean observed, typical individual predicted (PREDs) and individually predicted (IPREDs) concentrations, respectively.
