# Supplementary material for: A population pharmacokinetic model of cabozantinib in healthy volunteers and patients with various cancer types
Source: Cancer Chemother Pharmacol. 2018 Apr 23;81(6):1071–82. doi: 10.1007/s00280-018-3581-0 (PMC5973963; doi:10.1007/s00280-018-3581-0)
Supplement: Supplementary file 2 — Supplemental Fig. 2 Posterior Predictive Check: 90% Prediction Intervals and Observed Geometric Means, 10th and Percntiles of Cabozantinib Concentrations versus Time Profiles by Study for Patients with MTC. Squares, circles, and triangles correspond to observed median, 10th, and 90th percentiles, respectively. Middle, lower, and upper shaded areas correspond to 90% prediction intervals for median, and 10th and 90th percentiles, respectively. MTC medullary thyroid cancer (DOCX 111 KB) [file 280_2018_3581_MOESM2_ESM.docx]

**Supplemental Fig. 2**


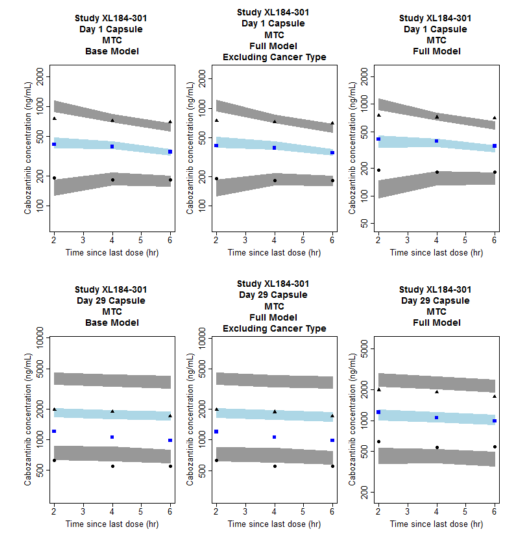


**Posterior Predictive Check: 90% Prediction Intervals and Observed Geometric Means, 10^th^ and Percntiles of Cabozantinib Concentrations versus Time Profiles by Study for Patients with MTC.** Squares, circles and triangles correspond to observed median, 10^th^, and 90^th^ percentiles, respectively. Middle, lower and upper shaded areas correspond to 90% prediction intervals for median, 10^th^ and 90^th^ percentiles, respectively. *MTC* medullary thyroid cancer
