# Supplementary material for: A population pharmacokinetic model of cabozantinib in healthy volunteers and patients with various cancer types
Source: Cancer Chemother Pharmacol. 2018 Apr 23;81(6):1071–82. doi: 10.1007/s00280-018-3581-0 (PMC5973963; doi:10.1007/s00280-018-3581-0)
Supplement: Supplementary file 3 — Supplemental Fig. 3 Visual Predictive Check for Cabozantinib Concentrations in Patients with MTC Using Full Model Re-Fit Including Only day 1 Data. Squares, circles, and triangles correspond to observed median, and 10th, and 90th percentiles, respectively. Middle, lower, and upper shaded areas correspond to 90% prediction intervals for median, and 10th and 90th percentiles, respectively. MTC medullary thyroid cancer (DOCX 54 KB) [file 280_2018_3581_MOESM3_ESM.docx]

**Supplemental Fig.3**


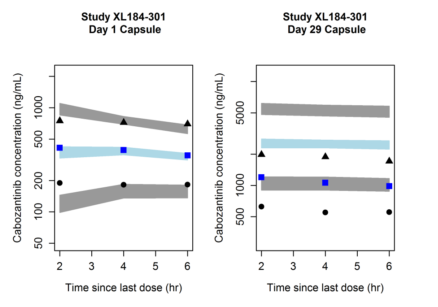


**Visual Predictive Check for Cabozantinib Concentrations in Patients with MTC Using Full Model Re-Fit Including Only Day 1 Data.** squares, circles and triangles correspond to observed median, 10^th^, and 90^th^ percentiles, respectively. Middle, lower and upper shaded areas correspond to 90% prediction intervals for median, 10^th^ and 90^th^ percentiles, respectively. *MTC* medullary thyroid cancer
