# Supplementary material for: A population pharmacokinetic model of cabozantinib in healthy volunteers and patients with various cancer types
Source: Cancer Chemother Pharmacol. 2018 Apr 23;81(6):1071–82. doi: 10.1007/s00280-018-3581-0 (PMC5973963; doi:10.1007/s00280-018-3581-0)
Supplement: Supplementary file 4 — Supplementary material 4 (DOCX 17 KB) [file 280_2018_3581_MOESM4_ESM.docx]

**Supplemental Table 1. List of Key Steps in Development of the Population PK Model Based on Integrated Cabozantinib Data**

| **Model Run** | **Description** | **OFV** | **Comment** |
| --- | --- | --- | --- |
| FULL (full5) | - 2-Compartment with dual first order absorption - Capsule as covariate on Ka and overall oral bioavailability - Age, weight, gender, race and population (RCC, CRPC, MTC, GB and other mixed malignancies) on both CL/F and Vc/F | NA | Model run not completed due to excessive run time |
| FULL-Vc  (full7) | Relative to FULL,   - All covariates on Vc/F were fixed to 0 as in previous analysis, no covariate effects were found on Vc/F | -3968.1 | - Run time ~ 3.5 days - Inspection of GOF plots shows reasonable fit |
| FM  (04a) | Relative to FULL, major changes are:   - First order + zero-order absorption were used to replace dual first order absorption to reduce run time - Dose dependent k12 estimated   Covariates included:   - Capsule as covariate on Ka and overall oral bioavailability - Age, weight, gender, race and population (RCC, CRPC, MTC, GB and other mixed malignancies) on both CL/F and Vc/F | -4639.7 | - Run time ~ 1.5 days - Inspection of GOF plots shows reasonable fit |
| BASE  (base1) | Relative to FM,   - Covariates except for dose and capsule were excluded | -4238.504 | Reference base model for FM,  ΔOFV=401 (df=22), suggesting some significant covariate effects |
| FMECT  (base3) | Relative to FM,   - Cancer type covariates were excluded | -4333.885 | Relative to FM, ΔOFV=305.8 (df=10), suggesting some significant effects of cancer type covariates |
| *OFV* objective function value, *FULL* original full model, *FM* full modified model, *FMECT* full model excluding cancer type covariates, *Ka* absorption rate constant from the 1^st^ absorption depot, *Vc/F* apparent distribution volume of central compartment, *RCC* renal cell carcinoma, *CRPC* castration-resistant prostate cancer, *MTC* metastatic medullary thyroid cancer, *GB* glioblastoma multiforme, *NA* not applicable, *GOF* goodness-of -fit | | | |
